# Multilevel Factors Influencing the Requirement for Geriatric Nursing by Older Adults Living With HIV: A Cross-Sectional Study

**DOI:** 10.3389/ijph.2024.1606820

**Published:** 2024-10-16

**Authors:** Mei Li, Yue Luo, Jian Lan Ren, Yu Zheng, Roger Watson, Yan Hua Chen

**Affiliations:** ^1^ Department of Gynecology, The First Branch of The First Affiliated Hospital of Chongqing Medical University, Chongqing, China; ^2^ School of Nursing, Southwest Medical University, Luzhou, Sichuan, China; ^3^ Department of Cardiology, West China Hospital, Sichuan University, Chengdu, China; ^4^ Department of Anesthesiology, The Affiliated Hospital of Southwest Medical University, Luzhou, China; ^5^ Department of Rheumatism and Immunology, The Affiliated Hospital of Southwest Medical University, Luzhou, China; ^6^ Health and Social Care Faculty, University of Hull, Hull, United Kingdom; ^7^ Department of Nursing, The Affiliated Hospital of Southwest Medical University, Luzhou, China

**Keywords:** HIV, geriatric nursing, needs, social support, stigma

## Abstract

**Objectives:**

People living with HIV are aging. This study aimed to assess the factors influencing the requirements for geriatric nursing of older adults living with HIV (OALHIV).

**Methods:**

Convenience sampling was used to conduct a survey on the 295 OALHIV aged over 50 in Luzhou, China.

**Results:**

OALHIV had few needs for living care needs. Most people indicate a requirement for reducing medical costs. Regarding psychological comfort needs, disease privacy and confidentiality were the greatest requirement. Multivariable regression analyses found that social support had a positive influence on the requirements for geriatric nursing.

**Conclusion:**

It is necessary to provide more social support for OALHIV. Most importantly, China should incorporate OALHIV into national pension security plan, integrate various resources and improve social security for them.

## Introduction

The aging of people living with HIV (PLHIV) is becoming increasingly prominent. Antiretroviral Therapy (ART) prolongs the life expectancy of PLHIV. In addition, there are more new people infected with HIV aged 50 and above, resulting in a growing number of older adults living with HIV (OALHIV; aged ≥50 years). According to UNAIDS in 2021, by 2020, there were 8.1 million older people aged 50 and above, accounting for about 21.5% of all PLHIV. China CDC Weekly reported [1] that by the end of 2020, a total of 1,053,000 people were infected with HIV in China, and the proportion of newly reported HIV-positive cases among males aged 60 and above increased from 7.41% in 2010 to 18.21% in 2020. The proportion of OALHIV is increasing, indicating that the aging trend of PLHIV is accelerating. One study [2] pointed out that recent years have seen a greater proportion and detection rate of HIV/AIDS cases among the ≥50 age demographic compared with the 15–49 age group in China. This study mentioned that the number of newly reported HIV/AIDS cases in the 15–49 age group increased from 51,436 in 2010 to 55,397 in 2022, while it increased from 11,751 in 2010 to 51,856 in 2022 in the group aged ≥50 years in China.

The international community has paid attention to the aging problem of PLHIV. In 2016, the 39th UNAIDS Conference focused on “HIV and Aging”, calling society to pay attention to the special needs of OALHIV. In China, according to the Regulations on AIDS Prevention and Control (revised in 2019), the national policy on AIDS focuses on prevention and control, treatment and assistance, and legal liability, but does not pay attention to the social security of OALHIV. In 2017, China State Council’s *13th Five-Year Plan for AIDS Containment and Prevention* proposed to strengthen the connection of relevant social welfare, social insurance, social assistance and other policies to ensure the rights and interests of PLHIV, such as basic medical care, basic pension, basic living and security.

However, OALHIV in China faces more challenges due to the social context. In China, sexual needs are often difficult to talk about, most people believe that older people do not have or need a sex life, and society’s attitude towards the sexuality of older people is predominantly negative [3]. One study of China pointed out that the public has a low understanding of older people with AIDS,and exhibits strongly negative attitudes, including discrimination and biases towards older AIDS patients [4]. Chinese scholars Feng Caiyun et al [5] found that family members discriminated against older HIV-infected patients and refused to take care of the patients, which forced older HIV-infected patients to live alone. According to Cantor’s Hierarchical Compensatory Theory of Social Support, the network fragility of OALHIV may made them seek care services [6]. Stigma is recognized as a barrier to the prevention, care, and treatment of HIV [7]. Social support facilitated their ability to engage in care, while stigma interfered with their ability to engage in care throughout the course of their illness [8].

Moreover, In addition, the health of OALHIV is not good. Studies [9, 10] found that, compared with older people without HIV infection, OALHIV can occur age-related complications more than 5 years earlier. They showed a higher incidence of co-disease and were prone to cardiovascular, liver, kidney, bone and other multi-system complications. One study [11] indicated that OALHIV increasingly needed formal supportive community services, such as medical and non-medical case management, clinical referrals, mental health, substance abuse treatment, housing assistance, legal services, nutrition, transportation, home care, emergency assistance, patient education support groups and other programs (AIDS Drug Assistance Program and secondary prevention services). Chayama et al. [12] pointed out that OALHIV had treatment and nursing needs. Kia et al. [13] conducted a qualitative study with 16 older gay men with AIDS, conceptualizing their service needs into practical, social and mental health needs; and the study pointed out that professional training should be provided to AIDS related nursing staff, so as to allow OALHIV to participate in and provide services for their own use.

Considering the aging trend in the PLHIV population and the difficulties they may face in old age, further understanding of their geriatric nursing needs is crucial. In addition, to promote the development of care services for older people specifically OALHIV, it is of great significance to carry out research on the geriatric nursing needs of this population. Therefore, this study aimed to investigate the geriatric nursing needs of OALHIV and identify the factors influencing the requirements for geriatric nursing, thereby providing data and evidence for the government to formulate care service policies for OALHIV.

## Methods

### Study Design and Participants

This study was a cross-sectional study, and judgement sampling was adopted in consideration of the concealment and privacy of AIDS. From May to November 2021, a questionnaire survey was conducted among OALHIV aged 50 and above who received follow-up management of antiretroviral therapy in the designated medical and health institutions for AIDS ART in Luzhou, China. Inclusion criteria of this study: (1) confirmed HIV-positive; (2) Age ≥50 years; (3) Those who had clear consciousness and able to communicate; and (4) Informed consent and voluntary participation in the study. First, we obtained permission from the regional CDC to sign a privacy and confidentiality agreement. We then conducted on-site questionnaire collection with the assistance of the community HIV follow-up manager. Eligible participants were recruited in the field and were instructed by the researcher to complete the questionnaire. Upon completion of the questionnaire, participants received some household items as gifts.

The sample size is calculated as a rule of thumb. A good general rule of thumb for factor analysis is 300 cases [14]. A total of 316 questionnaires were distributed in this study, and 295 valid questionnaires were obtained after removing invalid questionnaires, with an effective recovery of 93.4%. A preliminary survey was conducted with 29 OALHIV before the formal investigation, to evaluate the time required to fill in the questionnaire and the subjects’ understanding of the questionnaire, to check the feasibility and reliability of the survey method, and to find possible problems in the survey to reduce various biases. According to the problems existing in the preliminary survey, the final formal questionnaire was formed, and the formal survey was carried out. In order to avoid personal differences in understanding between the investigator and the elderly, we need to standardise the terminology used to explain the survey to the respondents and provide one-to-one guidance on completing the questionnaire. Therefore, before the investigation, we trained the investigators to unify the investigation methods and explanatory language.

### Measures

#### Definition of Concept

Different scholars have different definitions of demand. A need is a state of lack. Maslow [15] defined needs from the perspective of “humanistic psychology,” that needs are the intention and willingness expressed by the sense of lack, and ultimately produce human motivation.” There is no clear and unified definition of the concept of care needs of older people in the academic world. Based on Marx’s theory of humanistic needs, Zhang Na [16] defines it as the state of lack of physical, psychological, economic, cultural or social requirements of a person in old age. However, the aging of the body, and organ function degradation makes older people less mobile. This contributes to vulnerability, and the needs of the individual alone may be difficult to meet, requiring the help of others as well as the resources of society. Therefore, in this study, the concept of old-age needs was defined as the needs of older people for material and non-material help from other members of the society due to the changes in their physical, psychological and social living environment, which cause them to have relatively insufficient resources or even to be in a difficult situation in their old age [17].

The geriatric nursing needs are divided by Chinese scholars in terms of content and structure. According to the content of existing studies in China [18–20], geriatric nursing needs are mostly divided into three categories: living care, healthcare and psychological comfort, and each category can be divided into specific needs. Living care needs refer to the changes in people’s mental and physical conditions after entering old age, resulting in the need to rely on others to take care of their daily life, including subjective care needs (the desire to be cared for emotionally) and objective care needs (the need arising from the reality of physical difficulties). Healthcare, as a rigid need, is strongly demanded by older people due to the decline of their physical functions. Therefore, healthcare in this study refers to the provision of medical services. The need for psychological comfort refers to older people, especially OALHIV, whose negative events are more, psychological vacancy is likely to occur, so the heart has a strong demand for a happy and full life and a decent and dignified life, including personal emotions, social entertainment, communication with people, psychological support and other needs.

#### Measurement Tools

(1) Demographic information: individual situation (gender, age, education, household registration); family situation (marriage, whether living alone, number of children); economic situation (personal monthly income, refers to the average monthly income of the participants in RMB); and disease situation (length of diagnosis, chronic diseases, self-rated health, and HIV disclosure).
(2) Geriatric nursing needs were measured through a self-designed questionnaire containing 17 items in 3 dimensions: living care (5 items), healthcare needs (6 items) and psychological comfort needs (6 items). A five-point Likert scale was used to measure the degree of requirement (“not required, rarely required, neutral required, somewhat required, always required”). A score of 5 means that it is “always required”, a score of 4 indicates that it is “somewhat required”, and a score of 3 suggests that it is “neutral required”, 2 indicates “rarely required”, while 1 indicates “not required.” The range of scores is from 17 to 85. The higher the score, the greater the need for old-age care for OALHIV. Five experts engaged in AIDS, social medicine and geriatric nursing were invited to conduct two rounds of expert consultation. According to the pre-survey data, the internal reliability of the questionnaire was tested. The Cronbach’s α coefficient of the questionnaire was 0.897, indicating that the internal reliability was good. Five experts engaged in AIDS, social medicine and geriatric nursing were invited to conduct two rounds of expert consultation. Experts give opinions on the items, and content validity is determined quantitatively based on the expert’s rating of each item. Expert consultation results showed that S-CVI (Content Validity Index for Scales) and I-CIV (Content Validity Index for Items) were both 1.00.
(3) The social support scale developed by Chinese scholar Xiao was used to measure social support of OALHIV [21]. It has 10 items, including three dimensions, namely subjective support, objective support and use of social support. Subjective support refers to perceived social support, meaning that people feel supported, cared for, and helped by family members, friends and colleagues. Objective support refers to visible, practical and direct support. The utilization of support reflects the degree of social support used [22]. The scale is widely used and has good reliability and validity. In this study, the Cronbach’s α coefficient of this scale is 0.74. The score of total social support ranges from 12 to 66; a higher score indicates a higher level of social support. This scale was used to measure the social support of OALHIV and to explore the influence of social support on geriatric nursing needs. The Simplified Berger HIV Stigma Scale (SBHSS) was used to measure the internal stigma. The scale was derived from the Berger HIV Stigma Scale. Scholars from Peking University in China simplified the Berger scale and tested the reliability and validity of the SBHSS in 587 HIV-infected patients [23]. Cronbach’s α coefficient of the original scale was 0.78. The total score of HIV stigma was 15 points, and the higher the score was, the higher the stigma was. We used the scale to measure the internal stigma of OALHIV and to explore the influence of disease stigma on geriatric nursing needs.

### Analysis

First, demographic information was described by frequency and percentage. Social support and HIV stigma scores were statistically described by means and standard deviation. Second, in the statistical analysis, the five-point Likert scale was assigned a value of 1–5. The total score of geriatric nursing needs was obtained by adding the scores of 17 items of geriatric nursing needs. Based on the total score, participants were divided into three groups: those with low needs (17–39), those with medium needs (40–62), and those with high needs (63–85) for old-age care. T-test and analysis of variance were conducted using the three levels of need as the dependent variable. Finally, variables with P values < 0.2 in the univariate analyses were included in the multivariate ordered logistic regression analyses of geriatric nursing needs. P < 0.05 indicates statistically significant.

## Results

From Table 1, the gender was dominated by males (73.6%). The largest age group was 60–79 years old, accounting for 63.8%. The education level was generally low, among which 64.1% were primary school educated or below. More than half of those OALHIV were rural household registered (55.9%); 65.1% were married; people living alone accounted for 32.9%; 81.7% of older people had one or two children. From the income, 36.3% had a low personal monthly income of less than 1,000 yuan. From the perspective of disease conditions, those who had been diagnosed for 1–3 years accounted for the largest proportion (45.1%); 54.2% of OALHIV had one or more chronic diseases; in terms of self-evaluation of health, 58.3% of OALHIV believed that their current health status was good; and 78% of them had informed their family and friends of the condition. Most of OALHIV had medium needs. More details are shown in Table 1.

**TABLE 1:** Baseline characteristics and univariate analysis of demands for geriatric nursing (China, 2021).

Variable	Groups	n (%)	t/F	*p*-value
Gender	Male	217 (73.6)	−0.672	0.502
	Female	78 (26.4)		
Age (years)	50–59	95 (32.2)	0.204	0.893
	60–69	107 (36.3)		
	70–79	81 (27.5)		
	≥80	12 (4.0)		
Education	Elementary school or below	189 (64.1)	0.861	0.462
	Junior high school	75 (25.4)		
	Senior high school	24 (8.1)		
	Junior college and above	7 (2.3)		
Household registration	Urban	130 (44.1)	−2.299	0.022
	Rural	165 (55.9)		
Marital status	Married	192 (65.1)	1.386	0.167
	Divorced or widowed or unmarried	103 (34.9)		
Whether living alone	Yes	97 (32.9)	1.403	0.162
	No	198 (67.1)		
Number of children	0	15 (5.1)	0.550	0.648
	1	133 (45.1)		
	2	108 (36.6)		
	≥3	39 (13.2)		
Personal monthly income (yuan)	<1,000	107 (36.3)	2.606	0.036
	1,001–2000	83 (28.1)		
	2001–3,000	41 (13.9)		
	3,001–4,000	39 (13.2)		
	>4,000	25 (8.5)		
Length of diagnosis (years)	<1	58 (19.7)	2.280	0.079
	1–3	133 (45.1)		
	3–5	38 (12.9)		
	>5	66 (22.4)		
The number of chronic diseases	0	135 (45.8)	1.060	0.348
	1–3	149 (50.5)		
	≥4	11 (3.7)		
Self-rated health	Very poor	11 (3.7)	1.221	0.302
	Poor	40 (13.6)		
	Fair	72 (24.4)		
	Good	109 (36.9)		
	Very good	63 (21.4)		
Whether to inform friends and family of the condition	Yes	230 (78.0)	0.009	0.993
	No	65 (22.0)		
Social support	Total scores of social support	28.98 ± 7.011	1.950	0.002
HIV stigma	Total scores of HIV stigma	9.66 ± 0.193	1.010	0.445
Degree of needs	Low needs	69 (23.4)	-	-
	Medium needs	198 (67.1)		
	High needs	28 (9.5)		

Table 2 presents the geriatric nursing needs of OALHIV. In terms of living care needs, 15.9% of OALHIV needed housekeeping service and canteen service, 8.8% of OALHIV needed meals delivery services to the door, 8.5% of OALHIV needed goods purchasing agents, and 7.5% of OALHIV needed bill payment service. Of the healthcare needs, 82.7% had needs for reducing medical costs, 52.5% had needs for antiretroviral therapy guidance, 48.8% had needs for in-hospital care, and 41% had needs for emergency rescue. In terms of psychological comfort needs, a total of 92.9% of respondents needed disease privacy and confidentiality, 67.8% needed elimination of disease discrimination, 66.1% needed family care and companionship. The requirements for accompanying chat, cultural entertainment and psychological consultation accounted for 45.4%, 36.9%, and 15.3%, respectively.

**TABLE 2:** Descriptive analysis of geriatric nursing needs (China, 2021).

Requirement content	Degree of requirement				
	No needs (%)	Few needs (%)	Neutral (%)	Needs somewhat (%)	Needs very much (%)
Living care needs					
Housekeeping	172 (58.3)	63 (21.4)	13 (4.4)	30 (10.2)	17 (5.8)
Goods purchasing agent	200 (67.8)	57 (19.3)	13 (4.4)	16 (5.4)	9 (3.1)
Meal delivery service to the door	198 (67.1)	62 (21.0)	9 (3.1)	19 (6.4)	7 (2.4)
Bill payment service	198 (67.1)	59 (20.0)	16 (5.4)	15 (5.1)	7 (2.4)
Canteen for older people	166 (56.3)	70 (23.7)	12 (4.1)	24 (8.1)	23 (7.8)
Healthcare needs					
Reducing medical costs	2 (0.7)	4 (1.4)	45 (15.3)	92 (31.2)	192 (51.5)
Antiretroviral therapy guidance	19 (6.4)	59 (20.0)	62 (21.0)	69 (23.4)	86 (29.2)
In-hospital care	69 (23.4)	51 (17.3)	31 (10.5)	87 (29.5)	57 (19.3)
Emergency rescue	77 (26.1)	46 (15.6)	51 (17.3)	53 (18.0)	68 (23.1)
Home medical treatment	104 (35.3)	78 (26.4)	29 (9.8)	46 (15.6)	38 (12.9)
Medical escort service	124 (42.0)	74 (25.1)	23 (7.8)	45 (15.3)	29 (9.8)
Psychological comfort needs					
Disease privacy and confidentiality	3 (1.0)	5 (1.7)	13 (4.4)	40 (13.6)	234 (79.3)
Elimination of disease discrimination	14 (4.7)	18 (6.1)	63 (21.4)	75 (25.4)	125 (42.4)
Family care and companionship	26 (8.8)	33 (11.2)	41 (13.9)	74 (25.1)	121 (41.0)
Accompanying chat	55 (18.6)	60 (20.3)	46 (15.6)	88 (29.8)	46 (15.6)
Cultural entertainment	73 (24.7)	42 (14.2)	71 (24.1)	73 (24.7)	36 (12.2)
Psychological consultation	150 (50.8)	70 (23.7)	30 (10.2)	30 (10.2)	15 (5.1)

Multivariable analysis of the geriatric nursing needs (Table 3) showed that personal monthly income was negatively associated with the geriatric nursing needs. The higher the social support, the higher the rank of their old age needs. Participants who were divorced, widowed, or unmarried had more old-age needs.

**TABLE 3:** Multivariable ordered logistic regression analyses of demands for geriatric nursing (China, 2021).

Variables	Coef	95% CI	*p*-value
Monthly income	−0.260	−0.471–0.050	0.015
Diagnosis (years)	0.121	−0.114–0.357	0.313
Social support	0.076	0.034–0.117	<0.001
Household registration (Urban)	−0.304	−0.854–0.247	0.279
Marriage (Divorced or widowed or unmarried)	0.834	0.225–1.444	0.007
Living alone (No)	0.290	−0.323–0.903	0.354

## Discussion

OALHIV have less need for life care compared with ordinary older persons. On the one hand, it may be that they are now mostly living on their own and rely more on themselves or their spouses for life care; on the other hand, most of them come from rural areas, where there are fewer sources of economic income and lower incomes, and thus have less need for life care services that require spending money, which is like other studies [20]. In terms of healthcare needs, OALHIV had a lower need for medical convenience services (home medical treatment and accompanying to the doctor). Yet they had a higher demand for medical cost reduction, antiretroviral guidance, hospitalization accompaniment, and emergency care, which may be based on economic considerations, with medical cost reduction and satisfaction of important medical services as their main needs [20]. In terms of psychological comfort needs, OALHIV had the greatest need for privacy and confidentiality regarding their illness, which is consistent with the results of related studies [5]. Due to the stigmatization of AIDS, they worried that their family members and the public would not accept them and would be socially discriminated against if they knew about their disease. In addition, they also have a high demand for “elimination of disease discrimination” and “family care and companionship.” After being diagnosed with HIV, their internal stigma and external discrimination are intertwined, so they long for the understanding and care of their families and society and hope to eliminate disease discrimination and gain social respect. This suggests that society and medical personnel should pay attention to the mental health needs of OALHIV and help infected patients to reduce and eliminate the stigma of the disease, so that they can return to normal life.

Our results showed that social support had a positive impact on the geriatric nursing needs. The higher the level of social support, the higher the needs of the elderly. Social support theory [24] holds that the larger the social support network that individuals have, the stronger their ability to aggregate resources and satisfy their needs. Social support is divided into formal support (government, community, etc.) and informal support (family, friends, etc.). When OALHIV encounter difficulties, they usually seek help from close and trusted family and friends. The stronger their ability to seek help actively, the easier it is to acquire geriatric nursing services. Therefore, OALHIV with higher social support had higher demands. Zhong [25] pointed out that social support from family members and neighbors affected the requirement and use of health services for PLHIV. Cui et al. [26] showed that more than 90% of PLHIV hoped to receive in-hospital care from informal caregivers. In this study, the survey on social support of OALHIV found that the mean score of social support was lower than the national norm (M = 34.56, SD = 3.73) and the results [27, 28] on social support of PLHIV. This suggests that the government should fully mobilize the social support network resources of PLHIV and improve the use of social support resources by older people.

We found that individual monthly income negatively correlated with geriatric care needs. Requirement is mainly caused by the “unmet” reality. OALHIV with low monthly income have deficient old age resources, and their needs have not been met, so they have a larger requirement. For those with high monthly income, their geriatric nursing needs have been better met, so they have few needs. In this study, the OALHIV had a requirement for reduced medical costs. AIDS treatment is a long process, and the cost of treatment is particularly high. China provides free ART to PLHIV, but only certain free drugs are provided. Most AIDS patients do not need hospitalization, and 90% of their medical expenses are generated in outpatient treatment [29]. However, the outpatient expenses need to be paid by the patients themselves, which is a heavy burden for OALHIV with low income. In addition, AIDS-related health check-up costs and opportunistic infection treatment costs are high, which many patients cannot afford. All of these make it important to reduce the cost of healthcare for OALHIV.

We found that older people who are divorced, widowed or unmarried have a greater need for geriatric nursing. An article in The Lancet [30] points out that, at the level of Chinese society as a whole, the shrinking size of the family, the accelerating process of urbanisation and the increasing mobility of the population have led to the weakening of the traditional function of the family in providing for older people. The separation of parents and children has led to a “separation of support and care” and an increase in the number of empty nesters, left-behind and older people living alone. In many cases, many older persons spend their twilight years with their spouses providing mutual care and support. Older people who are divorced, widowed or unmarried lack the companionship and support of their spouses and therefore have greater needs in their old age. This suggests that the state and society should pay attention to OALHIV without spouses, who lack care support and have high old-age needs.

### Strengths and Limitations

We acknowledge that this study has both strengths and several limitations. One of the strengths is that it is one of the few studies investigating the requirement for geriatric nursing by older adults living with HIV. While globally rising clinical and research interests drive the move to promote and support OALHIV old-age care of OALHIV, few fundamental epidemiological studies have investigated the old-age care needs of this population. But as a cross-sectional study design, this study also has several limitations. First, a definite causal relationship is difficult to obtain by a cross-sectional analysis. Second, due to limited time and resources, the study was conducted in one city in China, having limited sample representativeness. In addition, the questionnaire for this study was self-designed and was not factor analysed at the time of design, nor did it take into account the difference between perceived and actual needs. In the future, more better scales could be developed to assess the perceived and actual needs of OALHIV. And multi-center large-scale research can be conducted in conjunction with other institutions to improve the generalization of data throughout China.

### Conclusion

Overall, most of OALHIV had moderate geriatric care needs. OALHIV had fewer requirements of living care. Their requirements for medical cost reduction and antiretroviral therapy guidance were high. They had considerable needs for disease confidentiality and elimination of disease discrimination. The requirement for geriatric nursing was influenced by social support. Hence support and care for OALHIV should be strengthened to reduce the inherent stigma and promote mental health. It is necessary to formulate and improve pension security strategies for OALHIV. Most importantly, China should incorporate OALHIV into the national pension security plan, integrate various resources and improve the social security level of this group.

## Data Availability

The original contributions presented in the study are included in the article/Supplementary Material, further inquiries can be directed to the corresponding author.
