# Corrigendum: Multilevel Factors Influencing the Requirement for Geriatric Nursing by Older Adults Living With HIV: A Cross-Sectional Study

**DOI:** 10.3389/ijph.2024.1608051

**Published:** 2024-11-14

**Authors:** Mei Li, Yue Luo, Jian Lan Ren, Yu Zheng, Roger Watson, Yanhua Chen

**Affiliations:** ^1^ Department of Gynecology, The First Branch of The First Affiliated Hospital of Chongqing Medical University, Chongqing, China; ^2^ School of Nursing, Southwest Medical University, Luzhou, Sichuan, China; ^3^ Department of Cardiology, West China Hospital, Sichuan University, Chengdu, China; ^4^ Department of Anesthesiology, The Affiliated Hospital of Southwest Medical University, Luzhou, Sichuan, China; ^5^ Department of Rheumatism and Immunology, The Affiliated Hospital of Southwest Medical University, Luzhou, Sichuan, China; ^6^ Health and Social Care Faculty, University of Hull, Hull, United Kingdom; ^7^ Department of Nursing, The Affiliated Hospital of Southwest Medical University, Luzhou, Sichuan, China

**Keywords:** HIV, geriatric nursing, needs, social support, stigma

In the published article, there was an error regarding the **Affiliations** of Mei Li. The author Mei Li has two affiliations.

Instead of “First Affiliated Hospital of Chongqing Medical University, Chongqing, China,” the correct affiliations were “Department of Gynecology, The First Branch of The First Affiliated Hospital of Chongqing Medical University, Chongqing, China” and “School of Nursing, Southwest Medical University, Luzhou, Sichuan, China.”

In the published article, there was an error regarding the **Affiliation** of Yue Luo.

Instead of “West China Fourth Hospital of Sichuan University, Chengdu, Sichuan, China,” the correct affiliation was “Department of Cardiology, West China Hospital, Sichuan University, Chengdu, China.”

In the published article, there was an error regarding the **Affiliation** of Jian Lan Ren.

Instead of “The Affiliated Hospital of Southwest Medical University, Luzhou, Sichuan, China,” the correct affiliation was “Department of Anesthesiology, The Affiliated Hospital of Southwest Medical University, Luzhou, Sichuan, China.”

In the published article, there was an error regarding the **Affiliation** of Yu Zheng.

Instead of “The Affiliated Hospital of Southwest Medical University, Luzhou, Sichuan, China,” the correct affiliation was “Department of Rheumatism and Immunology, The Affiliated Hospital of Southwest Medical University, Luzhou, Sichuan, China.”

In the published article, there was an error regarding the affiliation of Roger Watson.

Instead of “University of Hull, Hull, United Kingdom,” the correct affiliation was “Health and Social Care Faculty, University of Hull, Hull, United Kingdom.”

In the published article, there was an error regarding the **Affiliation** of Yanhua Chen.

Instead of “The Affiliated Hospital of Southwest Medical University, Luzhou, Sichuan, China,” the correct affiliation was “Department of Nursing, The Affiliated Hospital of Southwest Medical University, Luzhou, Sichuan, China.”

The **Corresponding author**’s * identifier was not identified on the correct name. Yanhua Chen is the corresponding author.

A correction has been made to the **Author Contributions** statement. The statement previously stated:

“All listed authors contributed significantly to the work. ML and YC conceptualized and designed the study. YL, JR, and YZ contributed to the data collection. ML, YL, and JR performed data analysis. ML and YZ drafted the manuscript. ML and YC contributed to the interpretation of results. ML, YL, JR, and YZ provided critical manuscript revision. All authors contributed to the article and approved the submitted version.”

The corrected statement appears below:

“All listed authors contributed significantly to the work. ML and YHC conceptualized and designed the study. YHC supervised the research. ML, YL, JLR and YZ contributed to the data collection. YL, RW and JLR performed the analysis and interpretation of initial data. ML drafted the manuscript. ML and YHC contributed to the final analysis and interpretation of the statistical results. RW embellished the language of the article. ML and RW revised it critically for the Discussion section of the article. All authors contributed to the article and approved the final published version. All authors agree to be accountable for all aspects of the work they are responsible for.”

The authors apologize for these errors and state that these do not change the scientific conclusions of the article in any way. The original article has been updated.

