# Supplementary material for: Multilevel Factors Influencing the Requirement for Geriatric Nursing by Older Adults Living With HIV: A Cross-Sectional Study
Source: Int J Public Health. 2024 Oct 16;69:1606820. doi: 10.3389/ijph.2024.1606820 (PMC11525981; doi:10.3389/ijph.2024.1606820)
Supplement: Supplementary file 1 [file DataSheet1.pdf]

The requirement for geriatric nursing of older people living with HIV

Dear Friend:

Greetings! This survey is targeted at people living with HIV aged 50 and above, aiming to understand your requirement for geriatric nursing. This survey is anonymous, and the relevant data will only be used for academic research, and will never be leaked, so we hope you will fill in the survey truthfully according to your own actual situation. Thank you for taking up your valuable time!

Instructions for filling in the form: Please use a ball-point pen, sign-pen or fountain pen to tick under the numbers in front of the options of each question, and fill in the corresponding contents on the horizontal line.

General information

1. Gender：①Male ②Female
2. Age: _________(years)
3. Education:

①Primary school and below ②Junior high school

③Senior high school ④Junior college and above

4. Household registration

①Urban ②Rural

5. Marital status

①Married ②Divorced

③widowed ④Unmarried

6. Whether living alone

①Yes ②No

7. Number of children: ___________
8. Personal monthly income (yuan)

①＜1000 ②1001-2000 ③2001-3000

④3001-4000 ⑤＞4000

9. Length of diagnosis: ____________ (years)
10. What chronic diseases do you have?

____

11. Self-rated health

①Very poor ②Poor ③Fair

④Good ⑤Very good

12. Whether informing friends and family of the condition?

①Yes ②No

The requirement for geriatric nursing

Requirement content	Degree of requirement				
	no needs	few needs	neutral	needs somewhat	needs very much
**Living care needs**					
Housekeeping					
Goods purchasing agent					
Meal delivery service to the door					
Bill payment service					
Canteen for older people					
**Healthcare needs**					
Reducing medical costs					
Antiretroviral therapy guidance					
In-hospital care					
Emergency rescue					
Home medical treatment					
Health care escort service					
**Psychological comfort needs**					
Disease privacy and confidentiality					
Elimination of disease discrimination					
Family care and companionship					
Chat-aid					
Cultural entertainment					
Psychological consultation					

Social Support Scale

1. How many close friends do you have that you can rely on for support and help? [Multiple choice] *

①0 ②1-2 ③3-5 ④6 or more

2. In the past year, you: [single choice] *

① Living away from family and living alone in one room.

② Living with strangers most of the time

③ Live with classmates, colleagues or friends.

④ Live with family

3. You and your neighbors: [multiple choice] *

① Never care about each other, just nodding friends

② May be a little concerned when faced with difficulties

③ Some neighbors: care about you

④ Most neighbors: care about you

4. You and colleagues: [Single choice] * (If no colleagues, villagers/community residents to judge)

① Never care about each other, just nodding friends

② May be a little concerned when faced with difficulties

③ Some colleagues: Very concerned about you

④ Most colleagues: very concerned about you

5. Support and care received from family members (tick "√" in the appropriate box) [table text question] *

	No	little	general	full support
Husband and wife (lovers)				
Parents (deceased _____ years?)				
Sons and daughters				
Siblings				
Other members (e.g. sister-in-law)				

6. In the past, the sources of financial support or help in solving practical problems that you have received when you were in an emergency situation are: [Single choice]
①No source ②The following sources (multiple choices allowed):
A. Spouse; B. Other family members; C. Friends; D. Relatives; E. Colleagues;
F. Work unit; G. Official or semi-official organisations such as party groups and trade unions; H. Unofficial organisations such as religious and social groups;
I. Others —________________(please list)

7. In the past, the sources of comfort and concern you have received in times of emergency have been: [Multiple choice]
①No source ②The following sources (multiple choices allowed):
B. Spouse; B. Other family members; C. Friends; D. Relatives; E. Colleagues;
G. Work unit; G. Official or semi-official organisations such as party groups and trade unions; H. Unofficial organisations such as religious and social groups;
II. Others —________________(please list)

8. The way you talk when you encounter troubles: [single choice] *
① Never confided in anyone

② Only talk to 1-2 people who are very close to you
③ If a friend asks, you'll tell them
④ Take the initiative to talk about your troubles in order to gain support and understanding
9. How to help you when you are troubled: [Single choice] *
① Only rely on themselves, do not accept help from others
② Rarely ask for help from others
③ Sometimes ask others for help
④ Often ask for help from family, relatives and friends, and organizations when in trouble
10. Regarding the activities organized by groups (e.g., party organizations, religious organizations, trade unions, student unions, etc.), do you: [Single-choice question] *
① Never participate ② Occasionally participate
③ Frequently participate ④ Actively participate and be active

The simplified Berger HIV Stigma Scale
1. Some people have distanced themselves from me after they learnt that I am HIV-positive.
①agree ②disagree
2. Most of the infected people are rejected when others know that they are infected.
①Agree ②Disagree
3. Most people feel uncomfortable knowing that there are people living with HIV around them.
①agree ②disagree
4. Most people think that people with HIV are disgusting.
①agree ②disagree
5. Having AIDS makes me feel unclean.
①agree ②disagree
6. Having AIDS makes me feel like a bad person.
①agree ②disagree
7. I don't feel as good as other people (not as normal as other people) because I am infected with AIDS
①agree ②disagree
8. I try my best to keep my HIV infection a secret
①agree ②disagree
9. I never feel the need to hide the fact that I am infected with HIV
①agree ②disagree
10. Instead of telling people that I am HIV-infected, I would rather not make new friends.
①agree ②disagree
The following questions assume that you have told others about your infection status, or that others know about your infection status, which may not be the case for you, please assume that you are in this situation)

11. Once people know I'm infected, they won't want me near their children
① I agree ② I disagree
12. Once people know I am infected, they will try to avoid contact with me
① I agree ② I disagree
13. I stopped associating with certain people because of their reaction to the situation
① I agree ② I disagree
14. I'm worried that someone who knows about me will tell someone
① I agree ② I disagree
15. I was hurt by people's reaction when they found out I had AIDS
① I agree ② I disagree
